# Innovating healthcare: Tangerine Clinic's role in implementing inclusive and equitable HIV care for transgender people in Thailand

**DOI:** 10.1002/jia2.26405

**Published:** 2024-12-23

**Authors:** Rena Janamnuaysook, Ratee Taesombat, Joe Wong, Ravipa Vannakit, Stephen Mills, Maarten Schim van der Loeff, Peter Reiss, Frits van Griensven

**Affiliations:** ^1^ Institute of HIV Research and Innovation Bangkok Thailand; ^2^ Amsterdam UMC Department of Global Health and Amsterdam Institute for Global Health and Development University of Amsterdam Amsterdam the Netherlands; ^3^ Amsterdam Institute for Immunology & Infectious Diseases Amsterdam the Netherlands; ^4^ Center of Excellence in Transgender Health Chulalongkorn University Bangkok Thailand; ^5^ Foundation of Transgender Alliance for Human Rights Bangkok Thailand; ^6^ Faculty of Tropical Medicine Mahidol University Bangkok Thailand; ^7^ Asia Pacific Transgender Network Bangkok Thailand; ^8^ BIRD – Bangkok Interdisciplinary Research and Development Bangkok Thailand; ^9^ Amsterdam Public Health Global Health Amsterdam the Netherlands; ^10^ USAID EpiC Thailand Project, FHI 360 Bangkok Thailand; ^11^ Public Health Service of Amsterdam Infectious Diseases Amsterdam the Netherlands; ^12^ Department of Epidemiology and Biostatistics University of California at San Francisco San Francisco California USA

Globally, anticipated and experienced stigma and discrimination against people with transgender and intersecting social identities in healthcare settings are barriers to HIV service uptake across the continuum of services [1]. This involves a lack of awareness, knowledge and understanding of transgender‐competent care among service providers. A consequence of this is low uptake of HIV services including adherence to antiretroviral treatment (ART) [2]. Also reported is arbitrary advice from healthcare providers involving a choice between ART and gender‐affirming hormone treatment (GAHT). Thus, there is a compelling case for establishing a transgender‐responsive health package across the HIV prevention, care and treatment cascade to meet their unmet health needs.

Despite the known high HIV prevalence in this population since 2010 [3], transgender women were not included as a distinct key population in Thailand's National AIDS Program. Categorized under men who have sex with men, they faced challenges accessing HIV services due to the lack of transgender‐inclusive care in public health facilities. In response, the Institute of HIV Research and Innovation (IHRI) established the Tangerine Clinic in Bangkok in November 2015—the country's first clinic to offer transgender‐competent HIV services.

In this Field Note, we share our experience implementing transgender‐competent HIV care at the Tangerine Clinic.

Our process involves four key components, illustrated in Figure 1.

**Figure 1:**
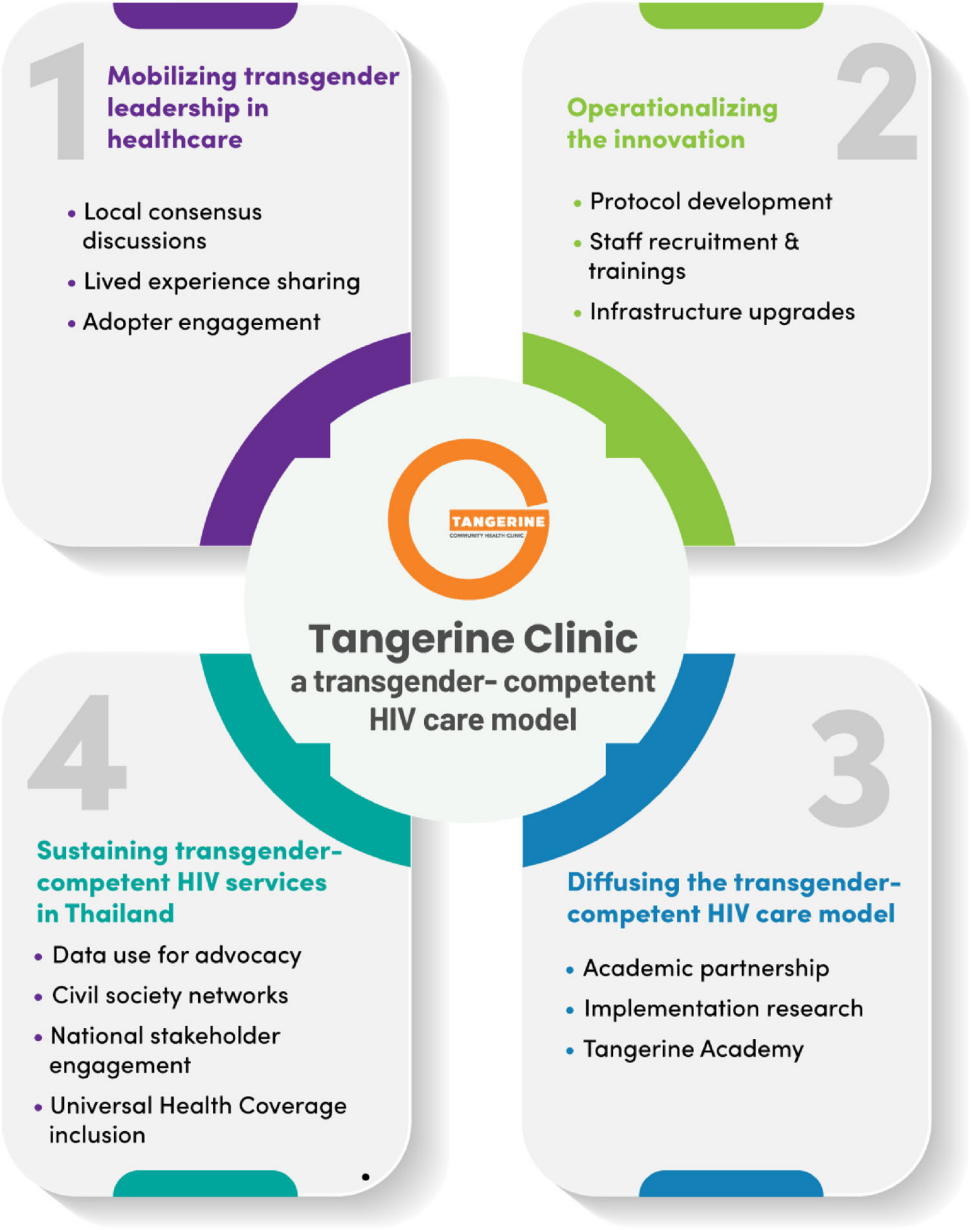
Key components in establishing and sustaining the Tangerine Clinic.

To promote transgender leadership in service design and delivery, IHRI facilitated a consensus meeting with the transgender community in Bangkok (September 2015). The consultation aimed to pilot a transgender‐competent HIV care model through community‐driven implementation research. Participants included a diverse range of transgender individuals, for example transgender women and men, sex workers, youth, persons with HIV, healthcare providers, academics and entrepreneurs.

Three key areas were identified: (i) *Health priorities* (GAHT, neovaginal care, HIV); (ii) *Essential service factors* (sensitive intake forms, stigma‐free providers, transgender‐inclusive healthcare team, confidentiality, safe space); and (iii) *Potential challenges* (lack of transgender‐competent physicians, invisibility of transgender men).

Dialogue between transgender delegates, providers and academics ensured the programme would be grounded in lived experience, scientific evidence and practicality. The consultation concluded with a consensus to establish Thailand's first transgender‐dedicated clinic, named “*Tangerine*” with the slogan “*where transition fulfills identities*.”

Reflecting the consensus achieved at the consultation regarding transgender health priorities, a protocol to deliver GAHT at Tangerine Clinic was developed. In parallel, the healthcare team undertook gender sensitization training and developed a comprehensive health service package, prioritizing GAHT as an entry point to HIV services. In addition to the transgender programme manager, two transgender women were recruited to the healthcare team as a clinic supervisor and peer counsellor.

In late 2015, funding from the United States Agency for International Development enabled Tangerine Clinic to procure clinic supplies and improve physical infrastructure to facilitate a safe space. Funding was earmarked to promote the clinic's branding and make communication materials available to transgender communities. Internally, IHRI's existing client records system was modified to accommodate preferred name and gender identities.

In 2016, Tangerine Clinic expanded its healthcare team by employing two additional transgender peer counsellors. The clinic subsidized laboratory testing for monitoring of hormone levels to supplement clients’ personal coverage of GAHT. To widen the reach of transgender communities, Tangerine Clinic involved transgender social influencers in recruiting clients from transgender sub‐populations [4]. Tangerine Clinic's annual number of clients grew from 446 in 2016 to 1050 in 2019 [5]. Between November 2015 and May 2023, the clinic has served 5939 transgender women, of whom 5396 (91%) received HIV testing; of those 471 (9%) were diagnosed with HIV, 440 (93%) of whom successfully initiated ART. Of those 440, 118 were referred to other long‐term ART maintenance facilities and we do not have data regarding viral suppression. Of the remaining 322 ART clients, 98% achieved viral suppression. The clinic has prescribed pre‐exposure prophylaxis (PrEP) to 1414 clients and post‐exposure prophylaxis to 554 clients, 254 (46%) of whom transitioned to PrEP as their prevention of choice, while 8 (0.56%) were seroconverted. Overall, HIV (*n* = 5396) and syphilis (*n* = 4583) testing and GAHT (*n* = 4001) were the most commonly used services by trans women, the latter of which was found to significantly increase subsequent clinic visits (*p* = 0.027), repeating HIV and syphilis testing (*p* = 0.020 and *p*<0.025, respectively) and PrEP use (*p* = 0.042) when compared to trans women who did not receive GAHT services [6].

In addition, Tangerine Clinic has served as IHRI's site for multiple clinical and implementation research concerning unmet transgender health needs, for example studies of drug‐drug interactions between GAHT and PrEP [7], self‐sampling to test for chlamydia and gonorrhoea [8], hormone concentrations [9] and the provision of mental health services. Fulfilling its goal to serve as a research‐to‐practice model, key study findings were used to inform the development of the clinic's standard of care. Tangerine Clinic currently provides a comprehensive range of transgender‐competent healthcare services tailored to the needs of its transgender clients. These services include testing, prevention, and treatment of HIV and other sexually transmitted infections, as well as the provision and monitoring of GAHT. The clinic also offers specialized neovaginal care, minor surgical procedures, and a variety of mental health and harm reduction interventions.

IHRI established the Tangerine Academy in 2017 as a regional technical assistance platform for scaling up transgender health programming in Thailand and the Asia region. The Academy serves to develop and share resources, including tools, training curricula, and provide clinical mentorship to support the rollout of transgender‐competent care models.

The Academy collaborated with the Health Department of the Bangkok Metropolitan Administration (BMA) to establish 31 Bangkok Pride Clinics within the BMA's Health Centers. To advance the science of transgender healthcare, the Tangerine Academy collaborated with Chulalongkorn University, to establish the Center of Excellence in Transgender Health. This resulted in Thailand's first operational guide on transgender healthcare services [10].

Tangerine Clinic has shown leadership in supporting the adoption of its transgender‐competent HIV care model. In 2023, the Academy trained 33 transgender lay providers to provide counselling on safe gender‐affirming hormone use in Thailand and five Asian countries. The model has been replicated in other Asian settings, including in Myanmar, the Philippines and Vietnam. The Tangerine Clinic also guided the development of the Asia Pacific Transgender Health Blueprint to strengthen the policy‐related, clinical, and public health responses on the provision of transgender‐competent care in the region.

To sustain transgender‐competent HIV services, Tangerine Clinic and its network of community‐led organizations submitted a proposal to the Ministry of Public Health to integrate transgender‐competent care into national HIV services. Such integration offers the dual benefit of safeguarding the right to gender‐affirming care and increasing access to HIV services among transgender communities through domestic financing. In December 2023, data from Tangerine Clinic were presented to the National Health Security Office for their healthcare benefit package review and consideration to include transgender‐competent care in the Universal Health Coverage (UHC) programme. Simultaneously, rights‐based civil societies conducted advocacy for transgender‐inclusive policies with parliamentarians. This successfully led to the government prioritizing support for transgender‐competent care which is expected to be incorporated into the national UHC programme in 2024. This will bring the country closer to ensuring inclusive and equitable healthcare for all transgender people and provide a model for other countries in the region.

## AUTHORS’ CONTRIBUTIONS

RJ conceptualized and developed the first draft of this Field Note. RV, MSVDL, PR and FVG provided guidance, input and subsequent revision. RT, JW and SM provided input and essential references. All authors have reviewed and approved the final manuscript.

## COMPETING INTERESTS

RJ received speaker's bureau fees and research grants through her organization from Gilead Sciences. RT, JW, RV, SM, MSVDL, PR and FVG declare that they have no competing interests.

## FUNDING

This work was supported by the United States President's Emergency Plan for AIDS Relief (PEPFAR) and the United States Agency for International Development Regional Development Mission for Asia (USAID RDMA) through Inform Asia project, Linkages across the Continuum of HIV Services for Key Populations Affected by HIV (LINKAGES) project (AIDOAA‐A‐14‐00045), and Meeting Targets and Maintaining HIV Epidemic Control (EpiC) project (7200AA19CA00002), led by FHI 360, and a grant from amfAR, The Foundation for AIDS Research, with support from the US National Institutes of Health's Fogarty International Center and the National Institute of Mental Health (CHIMERA; D43TW011302).

## DISCLAIMER

This work is solely the responsibility of the authors and does not necessarily represent the official views of any of the institutions mentioned above.

## Data Availability

Data sharing is not applicable to this article as no datasets were generated or analysed during the current study.
